# Bone of contention: Intra-element variability in remodelling of human femora based on histomorphometric and isotope analyses

**DOI:** 10.1371/journal.pone.0305089

**Published:** 2024-06-26

**Authors:** Yasmine A. de Gruchy, Katie E. Faillace, Katrien Van de Vijver, Eline M. J. Schotsmans, Jerrod Seifert, Adelle Bricking, Alexandra J. Nederbragt, Richard Madgwick

**Affiliations:** 1 School of History, Archaeology and Religion, Cardiff University, Cardiff, United Kingdom; 2 Royal Belgian Institute of Natural Sciences, Brussels, Belgium; 3 Centre for Archaeological Science, University of Wollongong, New South Wales, Australia; 4 Amgueddfa Cymru–Museum Wales, Cardiff, United Kingdom; 5 School of Earth and Environmental Sciences, Cardiff University, Cardiff, United Kingdom; University of Bern, Institute of Forensic Medicine, SWITZERLAND

## Abstract

The volume of human carbon (δ^13^C) and nitrogen (δ^15^N) isotope data produced in archaeological research has increased markedly in recent years. However, knowledge of bone remodelling, its impact on isotope variation, and the temporal resolution of isotope data remains poorly understood. Varied remodelling rates mean different elements (e.g., femur and rib) produce different temporal signals but little research has examined intra-element variability. This study investigates human bone remodelling using osteon population density and the relationship with carbon and nitrogen isotope data at a high resolution, focusing on variation through femoral cross-sections, from periosteal to endosteal surfaces. Results demonstrate considerable differences in isotope values between cross-sectional segments of a single fragment, by up to 1.3‰ for carbon and 1.8‰ for nitrogen, illustrating the need for standardised sampling strategies. Remodelling also varies between bone sections, occurring predominantly within the endosteal portion, followed by the midcortical and periosteal. Therefore, the endosteal portion likely reflects a shorter period of life closer to the time of death, consistent with expectations. By contrast, the periosteal surface provides a longer average, though there were exceptions to this. Results revealed a weak negative correlation between osteon population density and *δ*^15^N or δ^13^C, confirming that remodelling has an effect on isotope values but is not the principal driver. However, a consistent elevation of *δ*^15^N and *δ*^13^C (0.5‰ average) was found between the endosteal and periosteal regions, which requires further investigation. These findings suggest that, with further research, there is potential for single bone fragments to reconstruct in-life dietary change and mobility, thus reducing destructive sampling.

## Introduction

Due to differences in turnover rates, it is increasingly common to analyse stable carbon (*δ*^13^C) and nitrogen (*δ*^15^N) isotopes in several skeletal elements from an individual to elucidate diet during different periods of life [e.g., 1–3]. The adult rib and femur are most frequently sampled, as they are believed to be the fastest and slowest bones to turnover, respectively [e.g., 4–7]. However, in recent years, the validity of using the rib has been scrutinised, due to variability in isotope values between different ribs of the same individual and difficulty in identifying the rib number [8, 9].

In contrast to the numerous and wide-ranging studies on isotopes, variation in bone remodelling across the skeleton has received much less attention [see 4, 7, 10, 11] and our understanding remains equivocal. This is largely because it is incredibly difficult to study bone *in vivo* [12–16]. Access to skeletal collections of identified individuals is also limited, reducing the number of potential research subjects [11]. Despite this, it is generally acknowledged that the turnover rate of the human skeleton is 10% per year, based on an average of 4% per year in cortical bone and 28% per year in trabecular bone [17], though this is an over-simplified value based on limited empirical evidence.

Studies have shown discrepancies in observed remodelling patterns in the femur [e.g., 7, 10, 18, 19] and some suggest that certain areas within the bone may not remodel within a lifetime [e.g., 20, 21]. Moreover, despite being an essential premise for bioarchaeological isotope analyses, remodelling rates and intra-skeleton and intra-element variation remain under-researched and have to some degree become received wisdom. Given our limited understanding, this raises concern over the robusticity of using isotope analysis to reconstruct life histories and interpretations may be compromised. This paper examines an under-researched theme in studies on bone remodelling—intra-element variation, using the femoral cross-section (endosteal to periosteal), and how different approaches in sampling strategies may affect isotope values and, thus, interpretation.

### Remodelling rates and isotope analysis

In archaeology, the most cited bone remodelling study is Hedges et al. [4], which assessed the femur using radiocarbon tracer measurements of mid-shaft collagen in human individuals of known age and sex. Turnover rate in bone collagen can be analysed by exploiting atmospheric radiocarbon (^14^C) fluctuations caused by nuclear testing from 1950 to c. 1963, when atmosphere and underwater testing were banned [e.g., 4, 21, 22]. This ^14^C spike is used as a tracer of the year of carbon uptake in an organism during its lifetime. From their results, Hedges et al. [4] concluded that the turnover rate of bone collagen is faster in adult females (4% per year) than adult males (3% per year) and that turnover rate decreases with increasing age (down to 3% per year and 1.5% per year by the age of 80 in women and men respectively). They also observed varied residual ^14^C between individuals that could not be attributed to age or sex and instead credited to naturally occurring individual variation.

Very recently, a re-examination of the Hedges et al. (4) dataset, alongside cortical femoral ^14^C measurements from other published studies on ‘bomb pulse dating’ [23–25], was published by Quinn [26]. Using a different statistical approach than the original study, the remodelling rate was re-calculated to 3.5% per year. Moreover, Quinn [26] concluded there to be no significant difference in remodelling rates between females and males, and the changes with advanced age were found to be negligible. Whilst the two studies are not entirely comparable, Quinn [26] illustrates the value in building on from previous research, collating data and the importance of broadening the collective sample beyond a local population.

Fahy et al. [7] assessed cortical bone remodelling, in ten adult human individuals, on a wider range of skeletal elements, including the femur and rib, using a combination of stable isotope analysis (*δ*^13^C and *δ*^15^N) and a histomorphometric technique, known as osteon population density (OPD). OPD involves counting the frequency of secondary osteons and fragments within a region of interest (ROI). This was used as a proxy for bone remodelling, although the calculated value is expressed as the frequency of remodelling events, not as % per year as in Hedges et al. [4]. Bulk isotope analysis was carried out on the anterior quadrant of the midshaft of the femur, whilst OPD was carried out on the periosteal surface of the posterior portion.

Of the analysed elements, the humerus exhibited the highest turnover on average (15.10) and the occipital, the lowest (4.23) across both sexes. Meanwhile, the femur and rib displayed relative values of 13.48 and 13.90 respectively. When comparing the female data alone, however, the rib displays the highest turnover (15.98)—comparable with the humerus (15.89) and femur (15.60), among others. Nonetheless, these findings may have major implications for how multi-element isotope data are interpreted and how life-history research is designed, if elements considered to be two ends of the spectrum [e.g., 5, 6, 27] provide a comparable temporal signal.

Like Hedges et al. [4], Fahy et al. [7] reported that remodelling rates were typically higher in females than males. However, their results revealed a further interesting pattern: *δ*^15^N and remodelling were negatively correlated for eight of the ten individuals—for one individual this was significant (p = 0.007). Moreover, when all skeletons were combined, remodelling was significantly and negatively correlated with *δ*^15^N values (p = 0.050). No firm conclusion was offered for this, in part due to the small sample size. Lastly, Fahy et al. [7] propose that different elements with comparable OPD values may exhibit different isotopic signatures depending on the proportion of trabecular and cortical bone within the element, and that cortical bone surrounded by a higher index of trabecular bone may exhibit isotope values influenced by the trabecular value. This highlights the complex effects of turnover rates on isotope values and the need for more research on remodelling variation to inform sampling strategies.

Matsubayashi and Tayasu [21] explored variation in intra-element bone remodelling, by analysing the femoral midshaft of several faunal species, using *δ*^13^C and *δ*^15^N analysis and radiocarbon dating. Their results suggest that remodelling occurs at different rates along the direction of growth within the cross-section of cortical bone. Endosteal bone was found to contain the youngest radiocarbon values, before becoming rapidly older in the midcortical region and gradually younger towards the periosteal bone, reflecting periods during the bone’s growth. This has the potential to transform the way sampling for isotope analysis is carried out, as multiple phases could plausibly be extracted from a single element.

### Histological approaches to remodelling

Histological studies on intracortical remodelling in humans have primarily focussed on age-at-death estimation [e.g., 20, 28], although some have examined variability across different sections of limb bones [e.g., 18, 19, 29, 30]. These have demonstrated that remodelling occurs asynchronously across a single bone, varying concentrically and spatially. The leading explanation for these differences between bone quadrants is a disparate effect in response to mechanical strain and loading across the bone [29].

Using OPD, Gocha and Agnew [19] found that remodelling is typically highest in the periosteal regions of femoral midshaft bone, specifically the lateral and anterolateral areas, reported in almost all samples over the age of 35 (age range studied: 21–97 years at death). Their results further concluded that, on average, the endosteal third of the cortex had the lowest OPD values. This may be explained as cortical bone tissue being gradually removed from the medullary cavity in older persons [31]. A similar femoral study, between two individuals with estimated ages of 33 and 53, exhibited dramatically higher OPD values in the periosteal region of the older individual, concluding that a higher prevalence of bone remodelling units within the periosteal site is likely related to ageing [18]. By contrast, the radii from a group of individuals of a younger and smaller age range (n = 7, 25–35 years, 30) produced similar results of higher periosteal OPD values, suggesting that this is not an age-related pattern in the radius. These contrasting patterns across different elements demonstrate the equivocality of bone remodelling within the skeleton and calls into question the appropriateness of relying on different elements to explore dietary timelines.

To examine the systematic change in bone remodelling across the direction of growth in human femoral cortical bone, histomorphometric analysis (OPD) was carried out across the transverse section of the right femoral diaphysis of 20 individuals, comparing the endosteal, midcortical and periosteal regions. This was paired with stable *δ*^13^C and *δ*^15^N isotope analysis, using an adaptation of the collagen extraction method outlined by Matsubayashi and Tayasu [21], to determine whether temporal shifts in successive bone regions can be detected isotopically and whether a relationship between bone remodelling and isotope values exists. The overarching aim is to better understand intra-element variation in cortical remodelling, how this impacts isotope values and ultimately how this should guide sampling strategies and interpretations of past diet and mobility studies.

## Materials and methods

### Samples

Ethical approval was obtained in writing from the Research Ethics Committee of the School of History, Archaeology and Religion, Cardiff University, and sampling was carried out following the British Association for Biological Anthropology and Osteoarchaeology code of practice [32]. No permits were required for the described study, which complied with all relevant regulations. Human femur samples from 20 individuals (19 adults, 1 non-adult–SAK02) were obtained from the Department of Archaeological Heritage, Urban.Brussels. These derived from the parish cemetery of St. Anne, Koekelberg, Brussels, which was used between 1833 and 1916. Historical sources indicate a high proportion of working classes and poor in the parish population, although the use of zinc coffins also indicates the presence of middle and higher classes in the churchyard [33–35]. In 1985, remains from over 1000 graves were disturbed during redevelopment and the disarticulated bones were redeposited as commingled remains in the cellar of the sacristy on-site. In 2016, the disarticulated remains were collected during archaeological excavations and were made available for analysis. They have since been reinterred.

**Fig 1:**
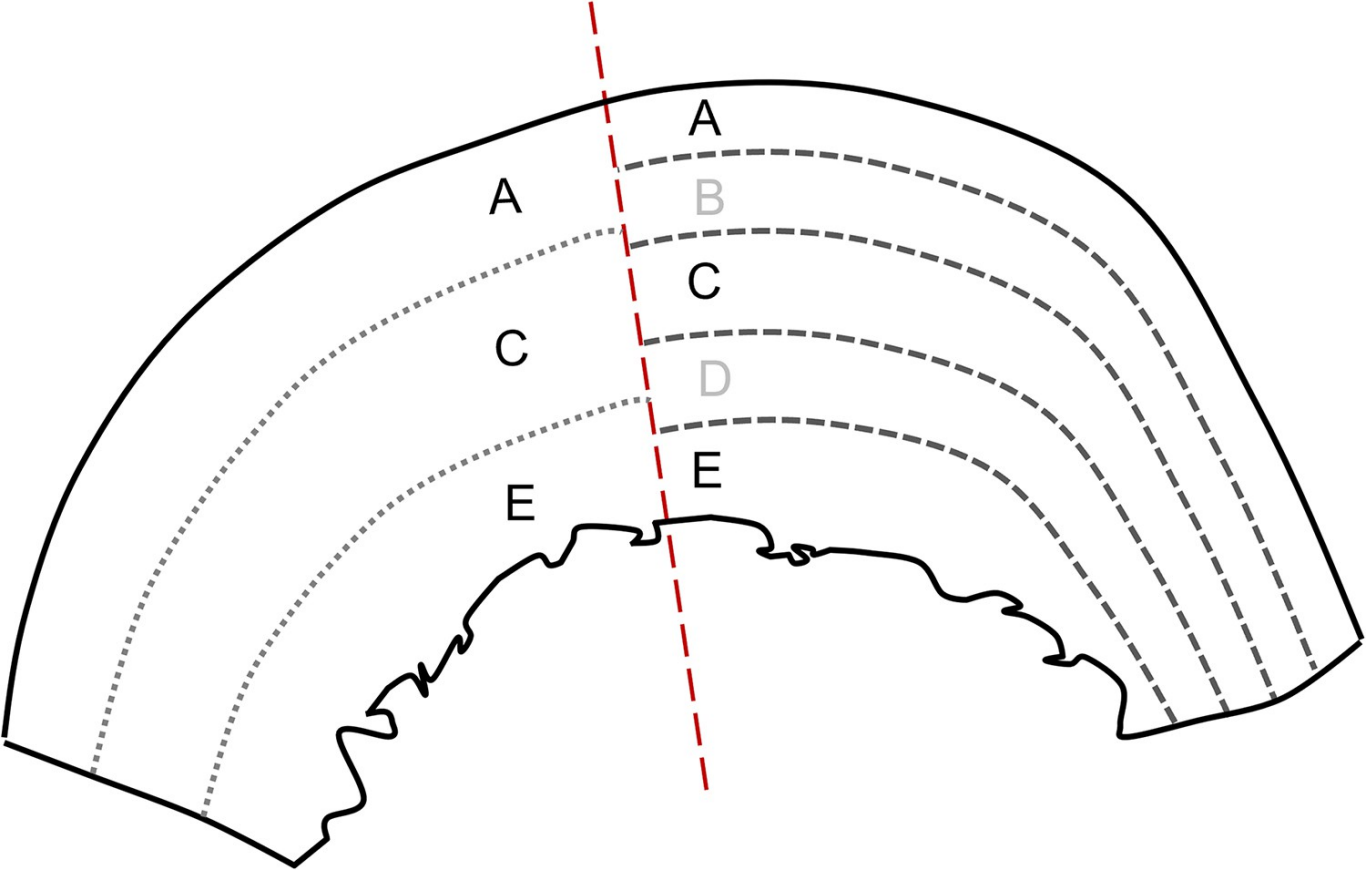
Schematic cross section of the anterior femoral midshaft, halved for OPD (left) and isotope (right) analysis, showing the divided sections and sampled segments: A (periosteal), C (midcortical) and E (endosteal). Segments B and D were not studied.

The femoral diaphysis was selected for study, as it is commonly targeted for isotope analysis and is the most researched element in bone remodelling studies [e.g., 4, 7]. The anterior midshaft portion was selected, as remodelling is less likely to be impacted by physical activity than, for example, the posterior portion. Therefore individual variation in lifeways (in terms of physical activity at least) should have a more limited impact on values [see 36]. Transverse sections (c. 10mm thick) from the periosteal to endosteal surfaces were extracted using a Dremel rotary tool with a diamond wheel attachment and divided into halves for isotope and histological study (Fig 1).

### Collagen extraction

Collagen extraction was undertaken at the Cardiff University BioArchaeology laboratory. The endosteal and periosteal bone surfaces were cleaned by mechanical abrasion (using a diamond burr) to remove adhering contaminants, trabecular bone and approximately 5–10μm of the cortex. The dry weight of each sample ranged between 1.1 and 3.6 grams.

Samples were immersed in 8ml of 0.5m HCl at 4°C for demineralisation. The acid was changed twice, and demineralisation was complete within two weeks. No NaOH pretreatment was undertaken. Once demineralised, the cross-sections were subdivided into five approximately equal transverse thin sections (labelled A to E; typically of 1.5–2.25mm thickness) from the periosteal (section A) to midcortical (C) and endosteal (E) surfaces using a scalpel (Fig 1). Hand-cutting was chosen over utilising a microtome [e.g., 37], as the thickness of the cortex was large enough to be easily divided by hand. Samples were then thoroughly rinsed with deionised water and gelatinised in pH3 H_2_O at 70°C for 48 hours. The remaining collagen solution was collected using an 8μm Ezee-filter and transferred to a polypropylene test tube. Samples were then freeze-dried using a Mechatech® LyoDry Compact freeze dryer. Once freeze-dried, all samples were weighed and the total weight of the five sections was used to calculate the collagen yield per individual. Next, 0.9mg (±0.2) of freeze-dried collagen was weighed in duplicate for each sample for *δ*^13^C and *δ*^15^N isotope analysis.

### Stable isotope analysis

Sections A, C, and E were selected for *δ*^13^C and *δ*^15^N analysis as these sections represent isolated periosteal, midcortical and endosteal bone respectively. Transitional sections were retained but not analysed. Collagen samples were analysed in duplicate using continuous flow-elemental analyser-isotope ratio mass spectrometry (CF-EA-IRMS) for *δ*^13^C and *δ*^15^N stable isotope composition. Isotope analysis was undertaken at the School of Earth and Environmental Sciences at Cardiff University using a Flash 1105 elemental analyser with ThermoFinnigan Delta V advantage. Carbon and nitrogen stable isotope ratios are given as *δ*^13^C and *δ*^15^N, relative to internationally defined standards Vienna Pee Dee Belemnite (VPDB) for carbon, and atmospheric air for nitrogen, expressed in units per thousand (‰). Stable carbon and nitrogen ratios were measured in accordance with international standards [38]. Atomic C:N ratios complied with collagen quality control criteria for nitrogen and carbon [39]. Carbon and nitrogen isotope ratios were calibrated against in-house caffeine (laboratory grade, 98.5%, Acros Organics, lot A0342883) and supermarket gelatine standards, which are calibrated against IAEA-600 (*δ*^13^C and *δ*^15^N), IAEACH-6 (*δ*^13^C) and IAEA-N-2 (*δ*^15^N). The 1σ (n = 55) standard reproducibility was ±0.06 for *δ*^13^C and ±0.07 for *δ*^15^N.

### Histological sample preparation and microscopy

The remaining bone was mounted using a two-component epoxy: EpoFix® Resin and EpoFix® Hardener and dried in a vacuum desiccator for a minimum of 12 hours. Once cured, thin sections were cut using an RMS-16G3 (REHA-Tech) microtome to a thickness of 60–70μm. These thin sections were mounted on glass slides using Entellan® rapid mounting medium.

Micrographs were taken under cross-polarised light using a Nikon Eclipse ME600 microscope with a SPOT RT3 microscope camera. OPD counting was undertaken using Fiji (a distribution of ImageJ) as follows. Images were obtained from two regions of interest (ROIs) per section (periosteal, midcortical and endosteal) from each femur, a total of six ROIs per individual. Each ROI was positioned adjacent to the periosteum and endosteum for the outer sections and central to the cortical thickness for the midcortical section. Intact secondary osteons and osteon fragments were counted in each ROI at a magnification of x50.

Features defining intact osteons and fragments were based on Haversian canal morphology as suggested by Stout and Paine [40] and defined by Heinrich et al. [41]. Secondary osteons were identified as containing an intact Haversian canal and the presence of a cement line. Secondary osteon fragments were defined as having the Haversian canal partially or completely removed by subsequent remodelling activity. These definitions were selected over alternatives [e.g., 28, 42–44] as these features are objectively distinctive. Osteons that had their Haversian canals overlapping across the border of the ROI were counted [45]. Intact and fragmentary osteons were both recorded as remodelling events and not individually recorded. This does not affect the outcome of the overall OPD. The OPD, and thus the evidence of past bone remodelling activity, was then calculated by dividing the total number of remodelling events by the area of the ROI (2mm^2^).

### Statistical analyses

Statistical analysis was carried out using R (version 4.0.3; R Core Team 2020). Differences in *δ*^13^C, *δ*^15^N and OPD between sections were tested separately using one-way ANOVA on ranks (Kruskal Wallis H test) followed by Bonferroni corrected Mann-Whitney U tests. Z-score standardised values for *δ*^13^C, *δ*^15^N and OPD were then analysed using linear regression analysis. We present the coefficient of determination (r^2^) and correlation coefficient (r) values, which measure the proportion of variation, strength, and direction of the relationship between isotope values and osteon density. The z-score data was then used to generate a generalised linear mixed model (GLMM). Bone section (A, C and E) was considered as the random effect variable to account for spatial pseudoreplication in the data.

## Results

**Fig 2:**
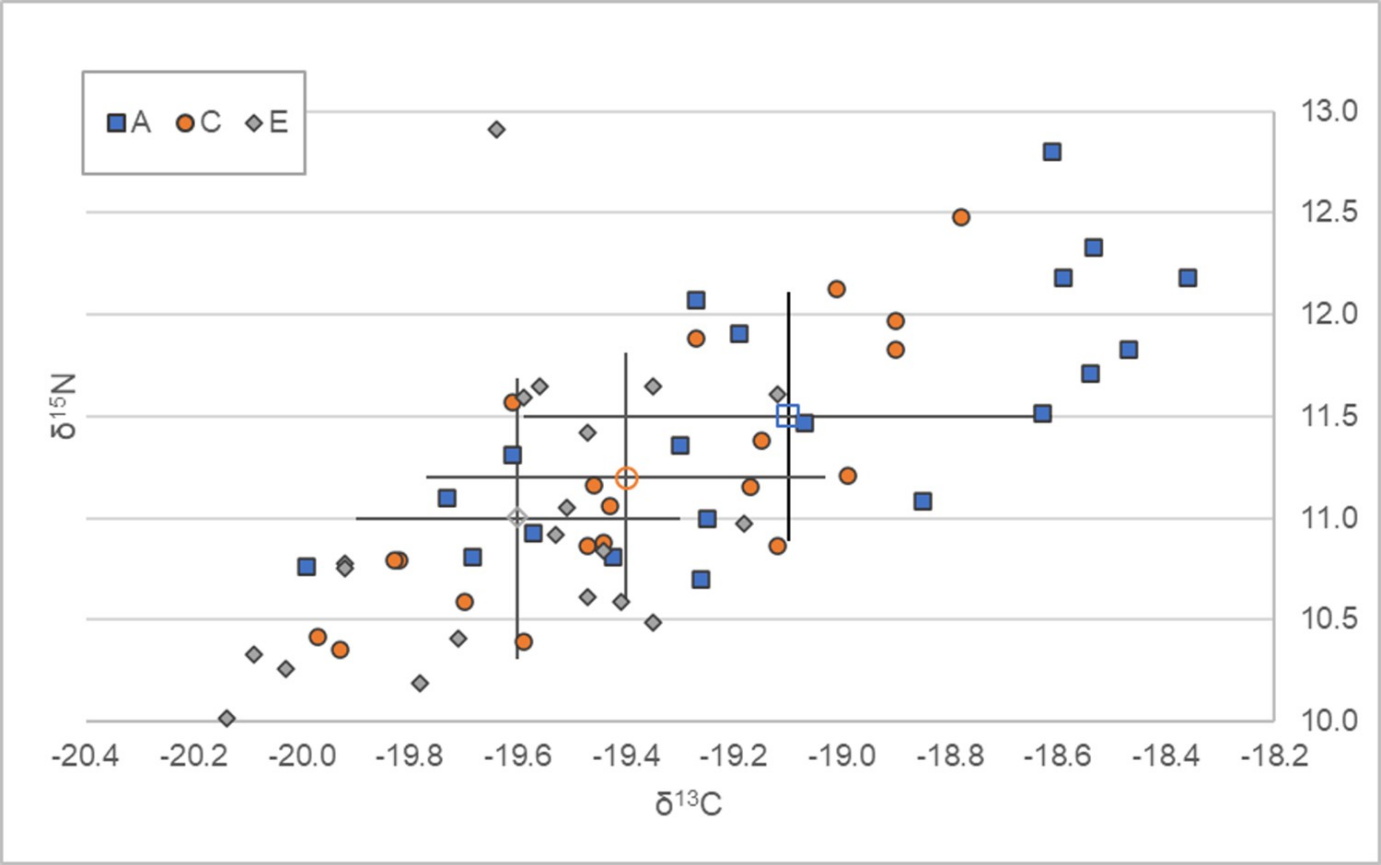
*δ*^13^C and *δ*^15^N isotope data for the different bone segments of all individuals.

**Fig 3:**
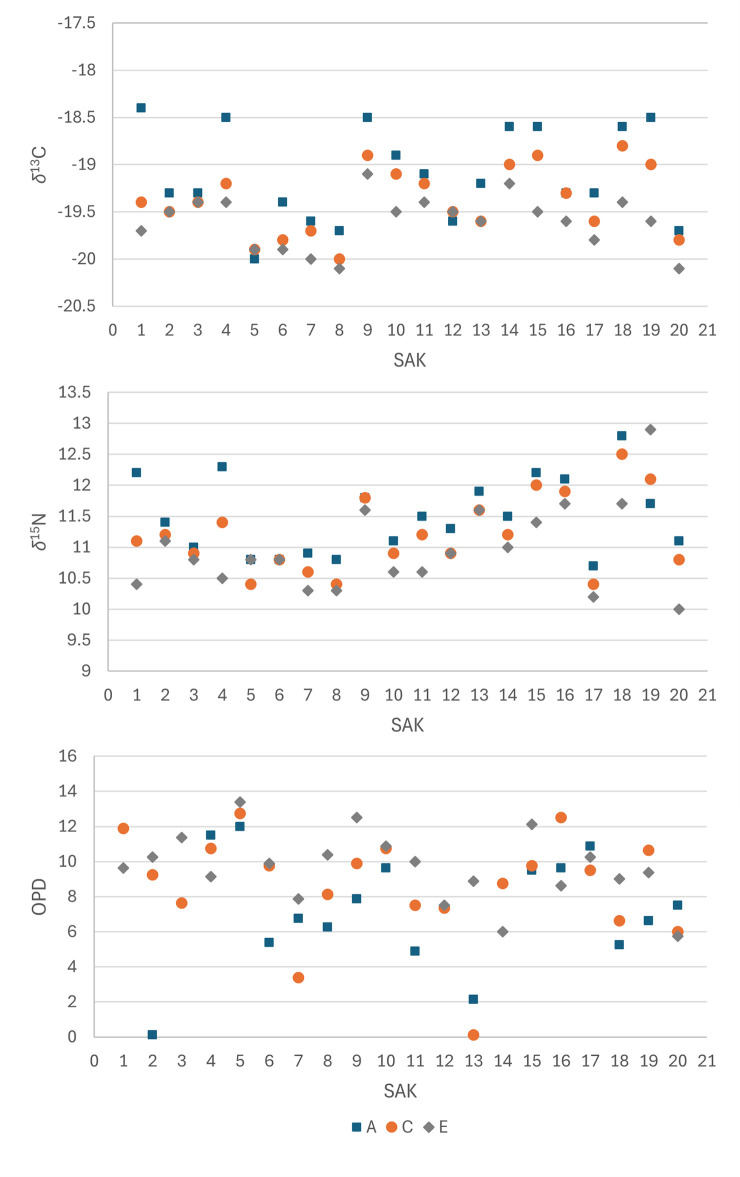
Individual variation of *δ*^13^C, *δ*^15^N and OPD across segments.

**Fig 4:**
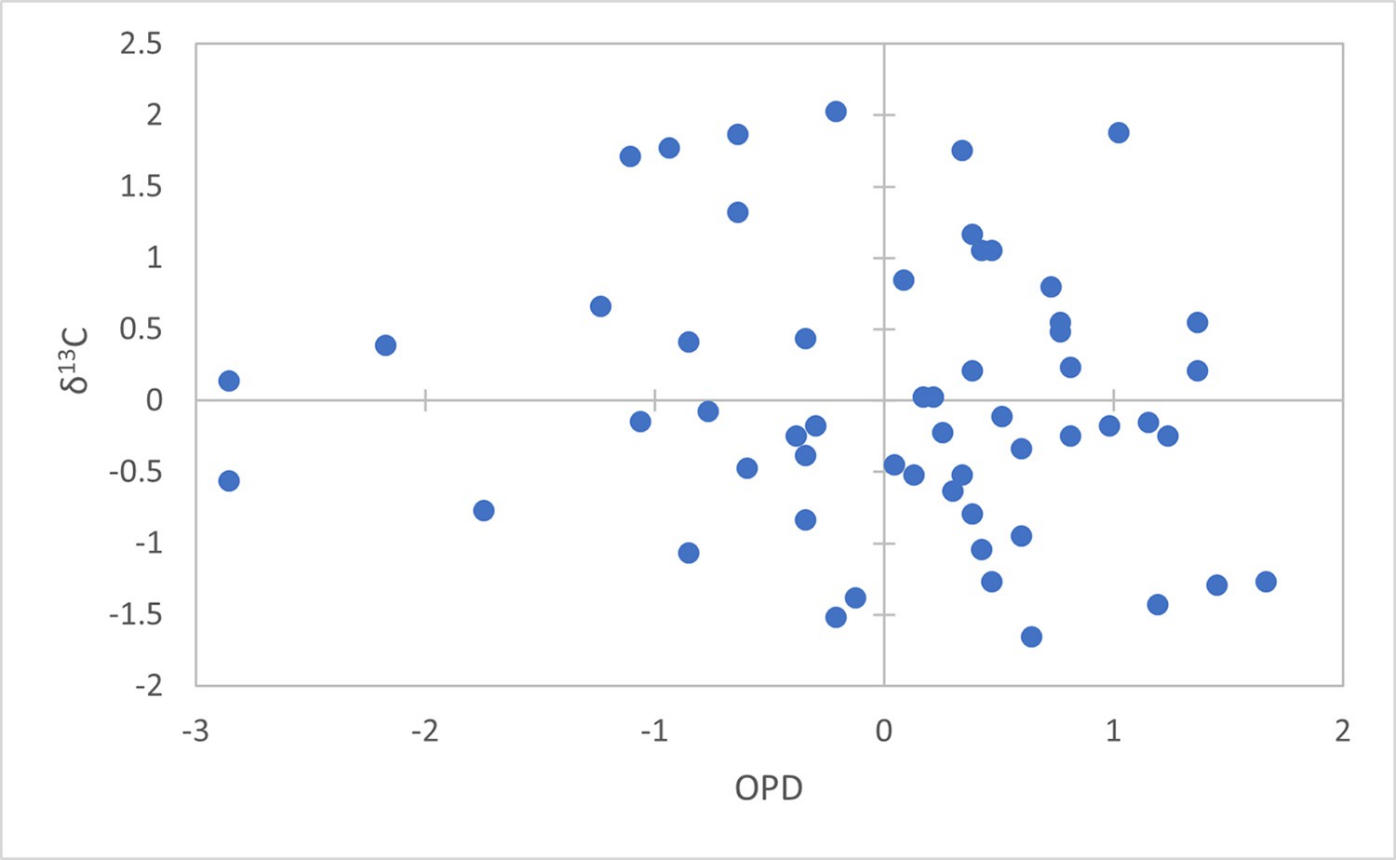
Comparison of z-normalised *δ*^13^C isotope and OPD data.

**Fig 5:**
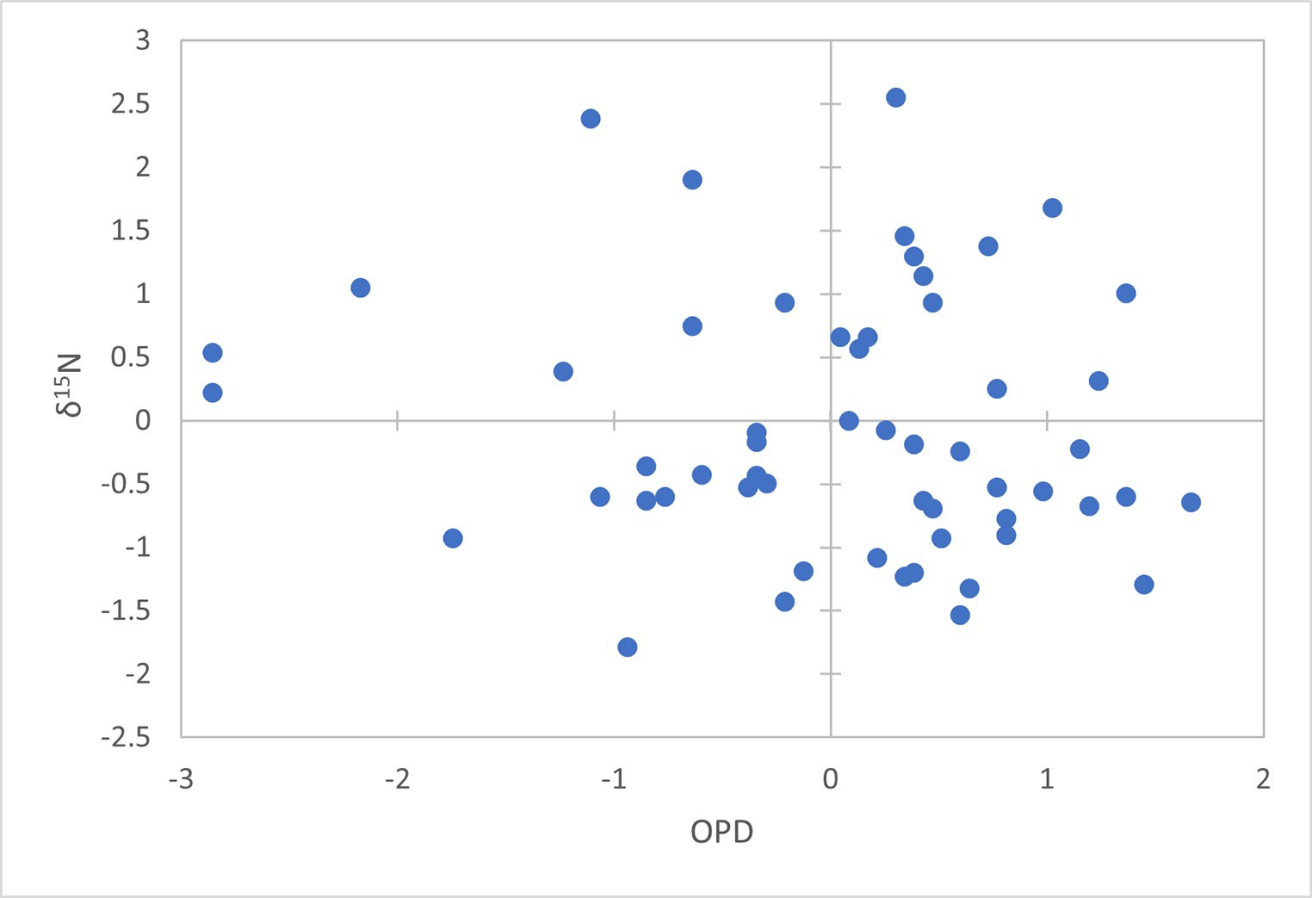
Comparison of z-normalised *δ*^15^N isotope and OPD data.

Results are presented in Tables 1 and 2 and–Figs 2–5 and described in the sections below.

**Table 1:** Isotope and OPD sample data.

Individual	section	δ^13^C	δ^15^N	%N	%C	C:N	OPD
SAK01	A	-18.4	12.2	15.36	41.72	3.2	-
	C	-19.4	11.1	14.66	39.95	3.2	11.88
	E	-19.7	10.4	15.82	43.46	3.2	9.63
SAK02	A	-19.3	11.4	16.56	44.72	3.2	0.13
	C	-19.5	11.2	16.69	46.06	3.2	9.25
	E	-19.5	11.1	16.30	44.14	3.2	10.25
SAK03	A	-19.3	11.0	16.50	44.74	3.2	-
	C	-19.4	10.9	14.86	41.12	3.2	7.63
	E	-19.4	10.8	16.88	47.29	3.3	11.38
SAK04	A	-18.5	12.3	16.37	44.42	3.2	11.50
	C	-19.2	11.4	16.17	44.49	3.2	10.75
	E	-19.4	10.5	16.05	44.83	3.3	9.13
SAK05	A	-20.0	10.8	16.41	45.24	3.2	12.00
	C	-19.9	10.4	15.41	42.71	3.2	12.75
	E	-19.9	10.8	15.69	44.61	3.3	13.38
SAK06	A	-19.4	10.8	16.14	43.78	3.2	5.38
	C	-19.8	10.8	16.06	43.51	3.3	9.75
	E	-19.9	10.8	15.63	43.87	3.3	9.88
SAK07	A	-19.6	10.9	16.60	44.68	3.1	6.75
	C	-19.7	10.6	16.52	45.15	3.2	3.38
	E	-20.0	10.3	16.30	45.42	3.3	7.88
SAK08	A	-19.7	10.8	16.19	43.84	3.2	6.25
	C	-20.0	10.4	16.04	43.96	3.2	8.13
	E	-20.1	10.3	15.59	44.98	3.3	10.38
SAK09	A	-18.5	11.8	16.30	44.42	3.2	7.88
	C	-18.9	11.8	16.38	45.00	3.2	9.88
	E	-19.1	11.6	16.45	46.45	3.3	12.50
SAK10	A	-18.9	11.1	16.04	43.48	3.2	9.63
	C	-19.1	10.9	15.01	41.09	3.2	10.75
	E	-19.5	10.6	15.37	43.27	3.3	10.88
SAK11	A	-19.1	11.5	16.02	43.56	3.2	4.88
	C	-19.2	11.2	15.92	43.66	3.2	7.50
	E	-19.4	10.6	15.72	44.46	3.3	10.00
SAK12	A	-19.6	11.3	15.30	41.75	3.2	-
	C	-19.5	10.9	26.38	44.36	3.2	7.38
	E	-19.5	10.9	26.07	45.13	3.3	7.50
SAK13	A	-19.2	11.9	16.66	44.96	3.2	2.13
	C	-19.6	11.6	16.34	44.83	3.2	0.13
	E	-19.6	11.6	15.88	44.72	3.3	8.88
SAK14	A	-18.6	11.5	16.49	44.60	3.2	-
	C	-19.0	11.2	15.92	43.08	3.2	8.75
	E	-19.2	11.0	16.45	46.24	3.3	6.00
SAK15	A	-18.6	12.2	16.51	44.78	3.2	9.50
	C	-18.9	12.0	15.61	42.80	3.2	9.75
	E	-19.5	11.4	16.15	45.27	3.3	12.13
SAK16	A	-19.3	12.1	16.43	44.15	3.1	9.63
	C	-19.3	11.9	15.39	41.94	3.2	12.50
	E	-19.6	11.7	16.19	45.25	3.3	8.63
SAK17	A	-19.3	10.7	15.98	43.19	3.2	10.88
	C	-19.6	10.4	16.49	45.35	3.2	9.50
	E	-19.8	10.2	16.78	47.21	3.3	10.25
SAK18	A	-18.6	12.8	16.73	45.01	3.2	5.25
	C	-18.8	12.5	16.48	45.06	3.2	6.63
	E	-19.4	11.7	17.17	47.87	3.3	9.00
SAK19	A	-18.5	11.7	16.72	44.94	3.1	6.63
	C	-19.0	12.1	15.52	42.72	3.2	10.63
	E	-19.6	12.9	16.02	44.96	3.3	9.38
SAK20	A	-19.7	11.1	16.09	43.32	3.1	7.50
	C	-19.8	10.8	15.72	42.93	3.2	6.00
	E	-20.1	10.0	15.49	43.56	3.3	5.75

**Table 2:** Summary statistics for OPD and isotope data in the three bone regions.

		A (periosteal)	C (midcortical)	E (endosteal)
**δ**^**13**^**C**	Median	-19.2	-19.4	-19.5
	Min-Max	-20.0 - -18.4	-20.0 - -18.8	-20.1 - -19.2
	IQR	0.86	0.54	0.38
	Mean	-19.1	-19.4	-19.6
	1SD	0.49	0.37	0.29
**δ**^**15**^**N**	Median	11.4	11.1	10.8
	Min-Max	10.7–12.8	10.4–12.5	10.0–12.9
	IQR	0.97	0.85	0.99
	Mean	11.5	11.2	11.0
	1SD	0.61	0.61	0.69
**OPD **	Median	7.13	9.38	9.76
	Min-Max	0.13–11.5	5.75–12.13	5.75–12.13
	IQR	4.32	3.19	1.69
	Mean	7.07	8.65	9.64
	1SD	3.24	3.04	1.96

### Preservation and taphonomy

All samples had an atomic C:N ratio between 3.1 and 3.3 and were consequently considered valid *δ*^13^C and *δ*^15^N values [46]. No abnormalities in *δ*^13^C or C:N associated with humic contamination were found across the samples [47, 48]. Furthermore, no staining was noted during macroscopic and microscopic evaluation, or at collagen extraction, ruling out the presence of humic acid within the samples [49, 50]. Histologically, preservation varied across the samples, though generally good, with intact Haversian canals and cement lines. However, for four individuals (SAK01, SAK03, SAK12 and SAK14) out of 20, the ROIs for the periosteal region were so heavily afflicted by diagenesis that OPD was not measurable (Table 1). As a result, the *δ*^13^C and *δ*^15^N values for these samples were omitted from linear regression analysis.

### Isotopic variation between bone segments

Isotope data showed limited variation. *δ*^13^C values varied between -20.1‰ and -18.4‰ and *δ*^15^N values from 10.0‰ to 12.9‰ across the three regions (Table 2).

*δ*^13^C and *δ*^15^N values typically increased (Fig 2) from the endosteal to periosteal regions, at an average of 0.5‰ for both isotope proxies, in all but three individuals (SAK05, SAK12 and SAK19; Fig 3) where the reverse was evident for either *δ*^13^C or *δ*^15^N. For 11 of the 20 individuals, this increase was gradual, with each progressive section exhibiting a different value. The periosteal region displayed the greatest range in *δ*^13^C values (-18.4‰ to -20.0‰), whereas the endosteal saw the greatest range in *δ*^15^N (10.0‰ to 12.9‰; Table 2 and Fig 3).

Results from the Mann-Whitney U test for each isotope suggest that there is no significant difference (using Bonferroni-corrected p<0.017) in values between the periosteal-midcortical (*δ*^13^C p = 0.0884 MWU: 137.0, *δ*^15^N p = 0.1636 MWU: 148.5) and midcortical-endosteal groups (*δ*^13^C p = 0.5310 MWU: 128.5, *δ*^15^N p = 0.1368, MWU: 145.0). However, the difference between the endosteal-periosteal groups was significant for both *δ*^13^C (p = 0.0007, MWU: 81.5) and *δ*^15^N (p = 0.0087, MWU: 103.0) with endosteal values being lower in both isotope proxies.

### Isotope variation within each individual

SAK01 displayed the largest difference between the endosteal and periosteal regions in both *δ*^13^C (+1.3‰) and *δ*^15^N (+1.8‰) values, implying a dietary or metabolic change. SAK05 showed the least variation between these two regions, with a difference of 0.1‰ for *δ*^13^C (-19.9‰ to -20.0‰) and no difference in *δ*^15^N (10.8‰). However, there was a difference in *δ*^15^N (0.4‰ decrease) between the midcortical region and the periosteal/endosteal regions in this individual.

No variation in either *δ*^13^C or *δ*^15^N isotope values between at least two adjacent regions was reported in seven individuals (Table 1), with SAK06 displaying no difference in *δ*^15^N values across all three sections of bone. Only SAK19 showed a gradual decrease in *δ*^15^N isotope values from the endosteal through to the periosteal surfaces (1.2‰). The same individual also displayed a considerable decrease in *δ*^13^C values between endosteal and periosteal sections at 1.1‰, potentially implying a small dietary or metabolic shift.

### OPD variation between bone segments

OPD values were wide-ranging across sections, from 0.13 to 12.13 across the dataset. The periosteal region had by far the greatest spread of values (0.13–11.50) and highest standard deviation (1σ = 3.29; Table 2). When the data for the 16 comparable individuals are combined, mean OPD values were highest in the endosteal region (9.64), followed by the midcortical region (8.65), and lowest in the periosteal region (7.07). The average difference in OPD values from the endosteal to periosteal regions was -2.60 per individual, whilst absolute variance ranged from -10.12 to +3.37. As with the isotope results, Mann-Whitney U tests revealed no significant difference between the periosteal-midcortical (p = 0.9563, MWU: 123), and midcortical-endosteal (p = 0.3720, MWU: 167) groups. However, the values from the endosteal region are significantly higher than the periosteal (p = 0.011, MWU: 93.5) which parallels the isotope data.

### OPD variation between individuals

OPD values and ranges varied markedly across individuals (Fig 3). Of the 16, nine showed a gradual decrease in OPD from the endosteal to periosteal regions. Meanwhile, SAK04 and SAK20’s values gradually increased across the same sections (+1.00 and +2.37 respectively). Together with SAK19, these three displayed lower OPD values in the endosteal cortex compared to the other regions. Whilst the midcortical OPD value was typically intermediate between the periosteal and endosteal values, this was not the case for six individuals. SAK07, SAK13 and SAK17 had lower OPD values in the midcortical segment compared to their preceding and succeeding regions. By contrast, the midcortical values for SAK01, SAK16 and SAK19 are the highest out of all three sections within these individuals.

SAK02, the single sub-adult, displayed by far the largest difference in OPD values from the endosteal to periosteal regions (-10.12), and the lowest periosteal OPD value in the dataset (0.13). The midcortical and endosteal regions are above average. No notable variation in isotope ratios was observed.

### Relationship between isotope values and bone remodelling

Of the 16 with complete OPD values, osteon density and *δ*^15^N are negatively correlated for 11 individuals. Further, there is a negative correlation between OPD and *δ*^13^C for 11 individuals, resulting in 10 individuals displaying a negative correlation for both isotope systems. A positive correlation between OPD and *δ*^13^C and *δ*^15^N was found for three individuals (SAK04, SAK17 and SAK20).

A linear regression analysis of z-scored data (n = 56; Figs 4 and 5) indicates that there is no significant relationship between OPD, and therefore bone remodelling activity, and either *δ*^13^C or *δ*^15^N (p = 0.33 and p = 0.38, respectively). However, the slope for both *δ*^13^C and *δ*^15^N suggests that there is a weak negative relationship (*δ*^13^C: slope = -0.127, r = 0.131, r^2^ = 0.017; *δ*^15^N: slope = -0.123, r = 0.120, r^2^ = 0.014). Results of the generalised linear mixed model also confirm that there is no relationship (p = 1) between the three variables when the bone regions are used as the random effect variable.

## Discussion

### Bone remodelling and isotope variation in cortical bone

The histomorphometric results in this study show that variation in bone remodelling exists across the transverse section of human bone. When all three regions are compared, the endosteal has the highest mean OPD, followed by the midcortical and periosteal. As higher osteon density indicates increased remodelling, the endosteal region has the highest proportion of reformed bone and potentially reflects an individual’s diet during a comparatively recent period prior to death. This may be explained by the role that endosteal bone plays in mineral homeostasis and the expansion of the medullary cavity during development and later life [51–53].

The femur gradually displays less remodelling along the direction of growth, with the periosteal region reflecting the ‘oldest’ bone (characterised by the least remodelling) present within the cross-section. The significant difference in OPD values between the periosteal and endosteal regions confirms that they reflect distinct (though overlapping) time spans. The significant difference in isotope values between these regions suggests the potential for using sequential sampling of femoral cortical bone to reconstruct an individual’s diet at multiple periods before death. However, these patterns appear variable, even if significant, and require further exploration to understand the timescales and variables at play.

To detect changes in diet or metabolism within an individual, DeNiro and Schoeninger [54] suggest a difference of at least 1.0‰ in *δ*^13^C and 1.4‰ in *δ*^15^N. However, incremental studies of dentine have shown smaller changes in individuals during different metabolic events [e.g., 55–57]. Only one individual, SAK01, surpassed the proposed limits, although some individuals displayed changes of at least 0.8‰ in *δ*^13^C and 1.0‰ in *δ*^15^N –markedly over the mean range (0.5‰ for both isotopes). These individuals likely also reflect a dietary or metabolic shift (e.g., illness, gestation, etc.), although establishing the aetiology of this shift in isotope values is beyond the remit of this research.

Linear regression revealed a weak relationship between OPD and either *δ*^13^C or *δ*^15^N, contrary to Fahy et al. [7] where OPD was significantly negatively correlated with *δ*^15^N values. The reasons for this difference require further investigation on larger and more diverse samples. That said, the consistent drop in isotope values (on average 0.5‰ for both proxies) between the periosteal and endosteal sections is a striking trend–suggesting another component impacts the isotope composition of cortical bone (e.g. transamination or other biological processes causing fractionation).

Proteomic investigations have highlighted the prevalence of non-collagenous proteins (NCPs) obtained from the ‘collagen’ extraction method used in stable isotope and radiocarbon analysis, and their distribution varies spatially within an element [46, 58–60]. Depending on their amino acid structure, these proteins may vary in isotope composition, particularly those with a higher proportion of non-essential amino acids, which fractionate across trophic levels [61]. Therefore, the consistent isotopic shift may reflect variation in protein composition across the bone samples, with the proportional contribution of NCPs differing from the endosteal to periosteal regions. This provides a fruitful avenue for future research and the impact of NCPs on dietary reconstruction should be investigated further, particularly in the light of the ever-increasing application of compound-specific isotope analysis which targets specific amino acids.

### Comparisons with previous research on inter- and intra-element bone remodelling

Whilst intra-individual bone remodelling variation and its effect on stable isotope values has been repeatedly investigated [e.g., 7–9, 54, 62], work on intra-element variation is relatively limited. Kontopoulos et al. [63] examined intra-element variability in bone collagen *δ*^13^C and *δ*^15^N isotopes in the proximal versus distal diaphysis of limb bones. No statistically significant difference in isotope values between the two regions was detected, except for one non-adult individual. By contrast, observations made on a single adult femur by Dauven et al. [9] presented a small difference in values between the quadrants within the transverse section of the midshaft (mean *δ*^13^C = 0.3‰, *δ*^15^N = 0.26‰) and much larger differences of up to 1.24‰ (*δ*^13^C) and 0.69‰ (*δ*^15^N) in other locations, including the diaphysis, the femoral head and neck. The contradictions between the studies demonstrate the complex nature of bone tissue and the need for further research.

With radiocarbon dating, Matsubayashi and Tayasu [21] were able to conclude that, in vertebrates, endosteal bone contains the youngest collagen, before becoming rapidly older in the midcortical section and progressively younger again towards the periosteal surface. Histologically, the authors state that whilst bone remodelling was primarily observed in the endosteal section, no evidence of remodelling in the other sections was present, meaning that the midcortical and periosteal zones likely reflect isotope values during skeletal growth in early life [21]. This broadly corresponds with our findings, with the periosteal region displaying the least remodelling, particularly in the case of SAK02, the non-adult with the lowest periosteal value in the dataset. Contrarily, three individuals (SAK04, SAK17 and SAK20) saw the highest OPD values in the periosteal portion. Studies imply a prevalence of remodelling in the periosteal region likely relates to ageing [18, 19], which is what we may be seeing here. These findings suggest that age is an important variable in dictating zonal remodelling and may explain some of the anomalies in this study. This possibility should be investigated further with samples from individuals of known age at death.

It seems that some degree of heterogeneity in bone remodelling between individuals is to be expected [4] and this is supported in the current study. In six individuals, the midcortical region displayed either the lowest or highest (both n = 3) OPD values within the transverse section. The reasons for these differences are uncertain, though possible interpretations could include unspecified pathological conditions, natural variation, or differences in age groups.

On several occasions, the intra-element variation of carbon and nitrogen isotope values exceeds proposed intra-skeletal limits for discriminating individuals in commingled remains. Berg et al. [62] suggest that two bones with *δ*^13^C isotope values differing by more than 0.75‰ or *δ*^15^N isotope values by more than 1.09‰ should be considered likely to derive from different individuals. Likewise, differences in *δ*^13^C values over 0.95‰ and *δ*^15^N over 1.35‰ were certain to belong to different individuals. It must be borne in mind that the criteria set by Berg et al. [62] relate to long bones, and not intended for use with other skeletal material, as data show that larger differences can be obtained between different bone types [e.g., 6, 7]. In this study there are 10 instances across six individuals where the absolute difference in intra-element values of either *δ*^13^C or *δ*^15^N was large enough to be considered either probably (n = 6) or certainly (n = 4) from a different individual. For these cases, this was up to 0.55‰ (*δ*^13^C) and 0.45‰ (*δ*^15^N) higher than the proposed limits for discriminating individuals, demonstrating that large differences can also derive from a single fragment of bone. We therefore suggest that sampling should be consistent and exercised with caution, comprising the entire cortical thickness taken from the same location, to avoid any potential zonal variation affecting the results.

### Contextual considerations: 19th century Belgium and nutritional stress

Cities in Belgium during the 19th century have been described in contemporary sources as ‘places of rampant disease, destruction and death’ [64]. This relates to numerous sociological factors, and average life expectancy at the time was just 40 years [65]. If higher instances of remodelling in the periosteal region are not prominent until after the age of 35 [19], it would be unlikely for this to be well-represented in a sample where many individuals may have died younger. That said, average life expectancy is greatly reduced by infant mortality and therefore some of the sample likely did reach beyond 40. Three individuals displayed the highest OPD values within the periosteal region and these may represent people of more advanced age.

Sustenance for the average working-class citizen across Belgium during the early 19^th^ century was based on a relatively homogenous diet with a high proportion of cereals and potatoes, and more limited supplements of dairy produce and particularly meat, with variation across regions and changes over time [66]. A restricted diet could account for limited variation in isotope data at both an intra- and inter-individual level. During the mid-19th century, Belgium saw several instances of harvest failures [66–68]. Malnutrition was likely a common affliction during this time, and it is likely that some individuals at the cemetery of St. Anne were undernourished, potentially causing differences in isotope values and remodelling.

**Fig 6:**
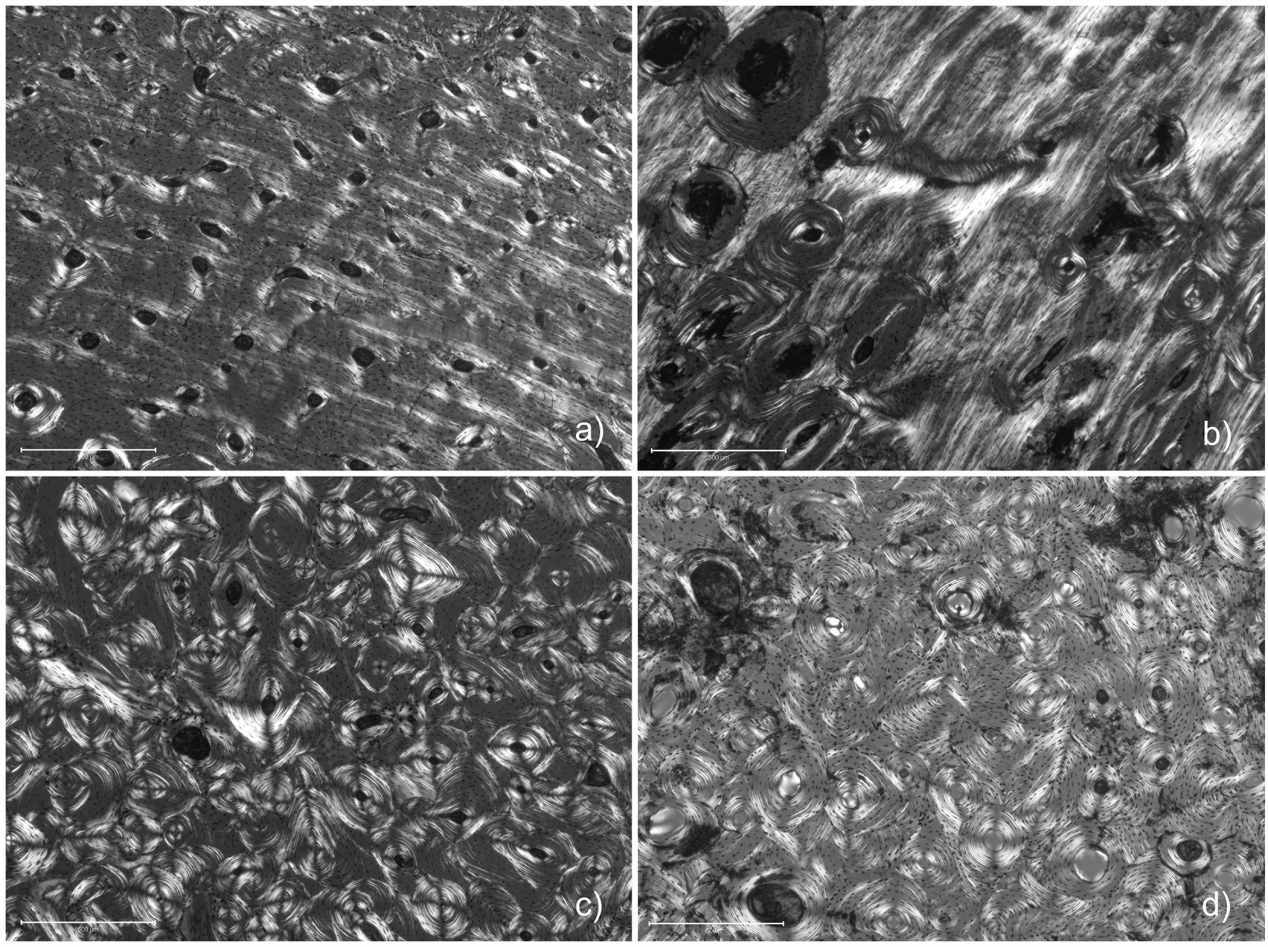
Micrographs displaying the midcortical region for SAK13 (a) and SAK07 (b), the two marked examples of possible malnutrition, compared with standard examples, SAK06 (c) and SAK19 (d) under polarised light.

There are several instances in this dataset where malnutrition may be in evidence, the most prominent examples being SAK07 and SAK13 (Fig 6). Both displayed primary lamellar bone with very few remodelled surfaces in either the periosteal and/or midcortical thirds of the cross-section and some of the lowest OPD values in the dataset (0.13 and 3.38 respectively for the midcortical region). These values are unlike SAK02 (OPD = 9.25), the single non-adult individual, which could rule out the possibility that SAK07 and SAK13 are closer in age-at-death to SAK02. Instead, these are likely malnourished individuals, as less remodelled bone (with fewer secondary osteons) can be indicative of nutritional stress [57, 69]. Interestingly, the endosteal OPD values for both SAK07 and SAK13, whilst below average, were not abnormal compared to the rest of the dataset.

Whilst bone growth is believed to arrest during periods of malnourishment in juveniles [57], in adults it can lead to catabolism of cortical bone tissue [70, 71]. Animal testing has revealed that this occurs predominantly within the endosteal bone [e.g., 52]. This is expected as the bone would be required to compensate for depleted mineral concentration of blood plasma caused by dietary deficiency and may explain why the endosteal OPD values for SAK07 and SAK13 are comparatively normal. Findings cannot determine whether these individuals were suffering malnutrition at time of death. However, the low frequency of secondary osteons in the outer portions show they sustained an extended period of nutritional stress, since youth. Limited histological research on the effects of nutritional stress and bone remodelling in humans has largely been carried out on, though not limited to, the rib [30, 69, 72–74]. Investigating the inter-element effect of malnutrition, metabolic disorders and its impact on isotope values is a profitable avenue for further research. Pathophysiological fractionation of *δ*^15^N in living tissues is a known effect of undernutrition [75]. Whilst detectable in non-remodelling tissues such as dentine collagen and hair [76–78] in bone collagen it is still poorly understood, although there has been some success in faunal studies [56, 57, 75, 79]. A small elevation in *δ*^15^N therefore may appear in tissues with low turnover rate [80].

### Research limitations

Our understanding of bone remodelling, its variation within an element and how it relates to carbon and nitrogen isotope values remains limited. The method employed in this paper is novel and there is therefore a paucity of directly comparable research. Given the limited nature of the dataset (right femora from 20 individuals from a single population), it is vital that similar studies are undertaken on more individuals and a wider suite of skeletal elements from different contexts to further elucidate the aetiology of variation in the different proxies. No assessment could be made on the potential relationship between sex, age-at-death and remodelling rate, as featured in previous research [e.g., 4, 7], due to the commingled nature of the remains sampled. Demographic information would have shed light on the individuals suspected of malnutrition and advanced age; previous studies have pointed to an increase in periosteal turnover in older individuals [18, 19], whilst another hypothesis that high protein diets may stimulate increased bone remodelling [81]. Limited research has been undertaken on the effect of malnutrition on histology across the skeleton.

OPD is not considered a suitable proxy for remodelling when dealing with bones from individuals of advanced age due to asymptote, where secondary osteon formation starts to remove past remodelling evidence [82, 83]. This is also problematic for bone regions where remodelling activity is more substantial. In this instance, the endosteal region likely reaches asymptote sooner than the midcortical or periosteal regions, which could explain the smaller interquartile range in the endosteal portion compared to other regions (Table 2). It is therefore worth considering utilising other histomorphometric features in tandem with OPD, such as lacunae/osteon geometry or osteon dispersal which are believed to be influenced by demographic factors [e.g., 84–87], to improve interpretive potential. In doing this, temporality may be better explored in samples where asymptote is reached.

## Conclusions

This study employs a novel method that advances the understanding of bone remodelling, intra-element variation and the relationship with carbon and nitrogen isotope values. The results demonstrate that remodelling varies between bone sections, occurring predominantly within the endosteal portion, followed by the midcortical with the least occurring within the periosteal bone. This indicates that endosteal bone undergoes rapid turnover during life and gradually slows, moving towards the periosteal surface. However, there were several instances where the periosteal region had the highest degree of remodelling, possibly related to the life history effects discussed above.

Statistical analysis demonstrates no significant relationship between either *δ*^13^C or *δ*^15^N and osteon population density (OPD) although a weak, negative correlation was shown between bone remodelling indices and *δ*^15^N. More interesting, however, is the consistency of lower endosteal values compared to periosteal values in both isotope proxies, suggesting the impact of another factor. One possibility that warrants further exploration is differing protein composition across the segments (dictated by varying proportions of non-collagenous proteins) and other processes causing fractionation including transamination. Whilst our results demonstrate a potential for utilising segmental isotope analysis of cortical bone for reconstructing life histories, more must be explored to support these findings using multiple lines of evidence.

Isotope analysis of bone has become a core method in archaeological research. However, reconstructing life histories has always been a challenge. Incremental sampling of dentine has allowed health and diet to be tracked through early life [88, 89], with recent improved approaches to micro-sampling providing greater temporal resolution [90, 91]. For later life, resolution is much more challenging, with comparisons of bulk collagen analyses of elements considered to have radically different remodelling rates often employed [often femur versus rib, e.g., 5, 76]. The segmental, intra-element approach employed in this paper could reduce the need to destructively sample multiple elements. This provides a more ethical approach to sampling, benefits cases where individuals are represented by few elements and helps preserve the valuable archaeological resource. It therefore unleashes new interpretative potential for commingled remains, which are often overlooked in scientific analysis.

Finally, the research demonstrates the notion of carefully designing and describing sampling strategies for collagen extraction (e.g., full transverse section of medial-midshaft) and preferably standardising them. Taking samples at inconsistent locations and depths within long bones could have substantial ramifications for results, in terms of temporality and absolute values. Direct comparisons of carbon and nitrogen isotope data must be made cautiously, with sampling strategies in mind. It is clear that the magnitude of intra-individual variation is frequently underestimated, and this variation occurs not only in different elements, or different parts of the same element but within a single fragment of an element. Sequential sampling across bone cross sections shows great promise, with the potential to gain more information about in-life dietary change without further destructive sampling.
